# Exercise echocardiography to assess left atrial function in patients with symptomatic AF^☆^

**DOI:** 10.1016/j.ijcha.2023.101324

**Published:** 2023-12-21

**Authors:** Jonathan P. Ariyaratnam, Ricardo S. Mishima, Olivia McNamee, Mehrdad Emami, Anand Thiyagarajah, John L. Fitzgerald, Celine Gallagher, Prashanthan Sanders, Adrian D. Elliott

**Affiliations:** Centre for Heart Rhythm Disorders, University of Adelaide, South Australian Health & Medical Research Institute and Royal Adelaide Hospital, Adelaide, Australia

**Keywords:** Atrial fibrillation, Left atrium, Strain, Exercise Tolerance, Symptoms

## Abstract

**Background:**

Left atrial (LA) function contributes to the augmentation of cardiac output during exercise. However, LA response to exercise in patients with atrial fibrillation (AF) is unknown. We explored the LA mechanical response to exercise and the association between LA dysfunction and exercise intolerance.

**Methods:**

We recruited consecutive patients with symptomatic AF and preserved left ventricular ejection fraction (LVEF). Participants underwent exercise echocardiography and cardiopulmonary exercise testing (CPET). Two-dimensional and speckle-tracking echocardiography were performed to assess LA function at rest and during exercise. Participants were grouped according to presenting rhythm (AF vs sinus rhythm). The relationship between LA function and cardiorespiratory fitness in patients maintaining SR was assessed using linear regression.

**Results:**

Of 177 consecutive symptomatic AF patients awaiting AF ablation, 105 met inclusion criteria; 31 (29.5 %) presented in AF whilst 74 (70.5 %) presented in SR. Patients in SR augmented LA function from rest to exercise, increasing LA emptying fraction (LAEF) and LA reservoir strain. In contrast, patients in AF demonstrated reduced LAEF and reservoir strain at rest, with failure to augment either parameter during exercise. This was associated with reduced VO_2Peak_ compared to those in SR (18.4 ± 5.6 vs 22.5 ± 7.7 ml/kg/min, p = 0.003). In patients maintaining SR, LAEF and reservoir strain at rest and during exercise were associated with VO_2Peak_, independent of LV function.

**Conclusion:**

The maintenance of SR in patients with AF is associated with greater LA reservoir function at rest and greater augmentation with exercise compared to patients in AF. In patients in SR, reduced LA function is associated with reduced exercise tolerance, independent of LV function.

## Graphical abstract

Summary of the study protocol and results. 105 patients satisfactorily completed exercise testing including CPET and exercise echocardiography. 31 presented in AF whilst 74 presented in SR. Patients in AF demonstrated reduced exercise capacity, reduced LA function at rest and failure to augment LA function with exercise. Reduced exercise capacity in patients with SR was characterised by reduced LA function at rest and with exercise, independent of resting LV function. **LV** left ventricle, **LVEF** left ventricular emptying fraction, **GLS** global longitudinal strain.

**Figure:**
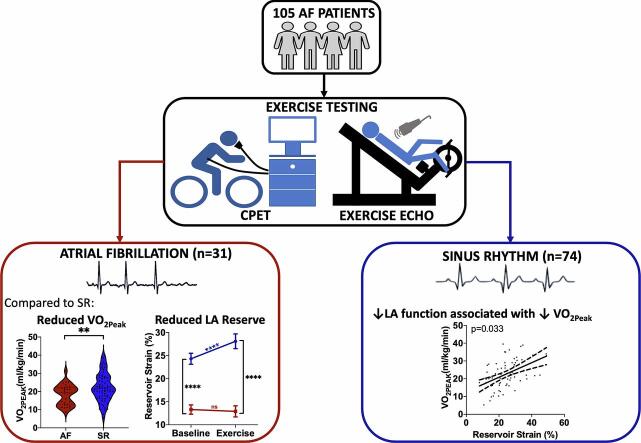

## 1 Introduction

Atrial fibrillation (AF) is the most common cardiac arrhythmia with an estimated worldwide prevalence of 53 million [1]. There is increased risk of mortality [2] as well as significant morbidity and reduced quality of life amongst patients with AF; 62 % of patients with AF demonstrate symptoms with 16.5 % experiencing severe or disabling symptoms [3]. Whilst palpitations are the most frequently reported symptom of AF, dyspnoea with exertion and exercise intolerance are highly prevalent and contribute significantly to reduced quality of life [3].

The mechanisms of exercise intolerance in AF remain poorly understood, particularly in patients with preserved left ventricular ejection fraction (LVEF) [4]. The loss of atrial systole during AF results in lower cardiac output and exercise tolerance. However, the atrial contribution to left ventricular (LV) filling during AF continues through its reservoir and conduit capacities. Left atrial (LA) reservoir function and emptying volumes are typically reduced in the presence of atrial disease and are associated with impaired exercise tolerance [5], [6]. Similarly, in patients with heart failure (HF), LA mechanical dysfunction at rest is associated with low exercise tolerance [7]. However, the data on how the LA responds to exercise during sinus rhythm and AF is limited.

Our aims were twofold; (i) evaluate the LA response during exercise amongst patients in SR and AF at the time of assessment, and (ii) amongst patients in SR, to evaluate the association between LA function during exercise and exercise tolerance. We hypothesised that reduced LA function with exercise correlates with reduced exercise capacity in patients with AF and preserved LVEF.

## 2 Methods

This prospective clinical study was undertaken at the Centre for Heart Rhythm Disorders (CHRD), University of Adelaide. The study protocol was reviewed and approved by the Human Research Ethics Committees of the Central Adelaide Local Health Network and the University of Adelaide. The study was prospectively registered with the Australian New Zealand Clinical Trials Registry (ACTRN12620000639921).

### 2.1 Study population

We prospectively recruited consecutive adult patients (>18 years) with symptomatic paroxysmal or persistent AF due to undergo an AF ablation procedure. Patients were excluded from participation for the following reasons: 1) reduced LV function (ejection fraction < 50 %), 2) prior diagnosis of a cardiomyopathy, 3) moderate-to-severe valvulopathy, 4) inability to perform cardiopulmonary exercise testing to completion, 5) inability to consent, 6) uncontrolled resting heart rate and 7) poor transthoracic echocardiography (TTE) imaging either at rest or during exercise.

### 2.2 Study design

All participants underwent cardiopulmonary exercise testing (CPET) for objective assessment of exercise capacity in addition to exercise echocardiography. Patients presented for CPET and exercise echocardiography on the same day. Exercise testing was performed in a fasting state and off rate-controlling and anti-arrhythmic medications (withheld for 48 h). Exercise echocardiography was performed first, followed by CPET. In order to assess the influence of cardiac rhythm on exercise capacity, participants were grouped according to the rhythm they presented with on the day of exercise testing (AF vs SR).

### 2.3 Resting and exercise TTE

Resting and exercise TTE were performed on a dedicated supine cycle ergometer in the left lateral decubitus position. TTE was performed according to a study specific protocol by an experienced sonographer. Images obtained included apical 4- and 2-chamber views and focussed on LV and LA structure and function. LV systolic function (LVSF) was measured by the Simpson’s biplane method for calculation of ejection fraction (LVEF). Maximum (LA_max_) and minimum (LA_min_) LA volumes were obtained using the biplane area-length method and were indexed according to body surface area. Left atrial emptying fraction (LAEF) was calculated using the formula: (LA_max_ − LA_min_)/LA_max_ x 100. Early LV filling velocities (E) using flow Doppler imaging and early diastolic mitral annular velocities (e′) using tissue Doppler imaging were measured. Average E/e′ was calculated as the mean of septal and lateral E/e′. Left atrial strain measurements using speckle tracking echocardiography were obtained offline using dedicated software (AFI LA, GE EchoPAC) according to standardised guidelines [8].

The exercise protocol involved cycling at a constant pedal speed of 60 revolutions per minute with incremental increases in power of 10 Watts/minute. Focussed LA and LV TTE images were obtained at peak exercise defined as the point of fusion of the E and A waves. LVEF, E/e′, TR V_max_, LV global longitudinal strain (LV GLS), LAEF and reservoir strain measurements were obtained at peak exercise. Booster and conduit strain were not assessed due to fusion of reservoir and booster components at peak exercise. All measurements were averaged over 3 cardiac cycles in sinus rhythm and 6 cycles in AF and verified by a second experienced and independent reviewer.

### 2.4 Cardiopulmonary exercise testing

Symptom-limited CPET was performed on a dedicated upright cycle ergometer. Twelve (12) lead ECG was attached and monitored throughout to assess heart rate and rhythm. Pulmonary gas exchange was measured continuously using a metabolic cart (Vyntus CPX, Vyaire Medical). Oxygen consumption (VO_2_) and carbon dioxide (VCO_2_) production were averaged over 20 s intervals and adjusted to body mass (ml/kg/min). Prior to exercise, participants underwent a 5-minute rest period to obtain baseline values. Participants were then asked to begin cycling at a power of 20 Watts. Power was incrementally increased by 10 Watts per minute. Peak exercise was defined as the point at which the participant felt the need to stop due to symptoms or fatigue. A maximal effort was defined as having reached a respiratory exchange ratio > 1.05. Peak oxygen consumption (VO_2Peak_) was identified as the highest attained VO_2_ during exercise. A VO_2Peak_ < 20 ml/kg/min (Weber Class B) was considered objective evidence of reduced exercise capacity as previously described [9].

### 2.5 Statistical analyses

Continuous variables were reported as means ± standard deviation for normally distributed data or median and interquartile range for non-normally distributed data. Categorical variables were reported as frequencies and percentages. Continuous variables were compared between groups using independent-samples Student t-tests or Mann Whitney U tests as appropriate. Categorical variables were compared between groups using the chi-square test or Fisher’s exact test. Comparisons between resting and exercise TTE parameters were made using paired-samples Student t-tests. Associations between VO_2Peak_ and exercise echocardiography parameters were assessed using adjusted linear regression models. Model 1 adjusted for age and gender, whilst model 2 adjusted for age, gender and resting LVEF. P-values of ≤0.05 were considered statistically significant. All statistical analysis was performed using R version 4.0.3 (R Foundation for Statistical Computing, Vienna, Austria).

## 3 Results

**Fig. 1:**
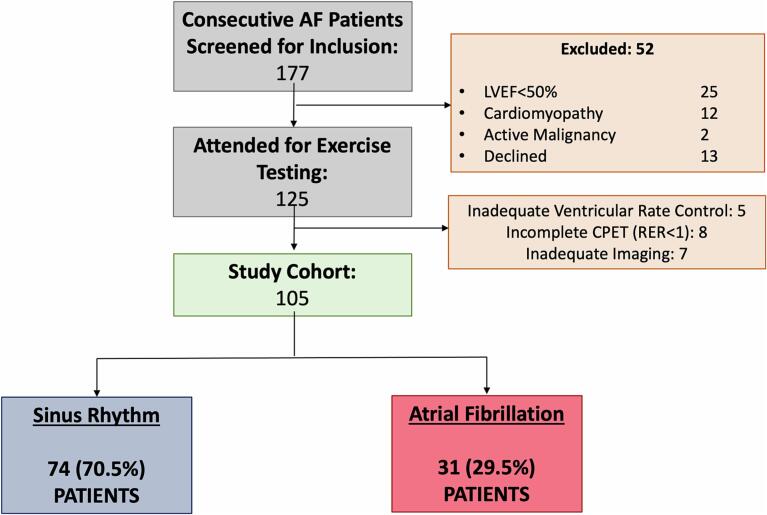
CONSORT diagram. Consort diagram showing patient recruitment and classification including reasons for exclusion. **LVEF** left ventricular ejection fraction, **RER** respiratory exchange ratio.

Of 177 consecutive symptomatic AF patients, 39 were excluded for pre-specified exclusion criteria and a further 13 declined to participate. The remaining 125 patients consented to undertake the protocol and presented for exercise testing. However, a further 20 patients were excluded from the analysis, due to inadequately controlled ventricular rate (n = 4), incomplete CPET (n = 8) or inadequate imaging during exercise echocardiography (n = 8). In total, therefore, 105 patients were included in the analysis with 74 presenting in SR and 31 presenting in AF (Fig. 1).

### 3.1 Baseline characteristics

Table 1 compares the patient demographics and baseline characteristics of patients presenting in SR and AF. Patients in AF were more likely to have persistent AF and take loop diuretics. There were no other significant differences in demographics, risk factors or medications taken between the 2 groups.

**Table 1:** Baseline Characteristics. Patient characteristics, comorbidities and medications across each presenting rhythm group (AF versus SR). Data are means ± standard deviations, medians (interquartile range) or numbers (%) of cases. **AF** atrial fibrillation, **BMI** body mass index, **ACEi** angiotensin converting enzyme inhibitor, **ARB** angiotensin receptor blocker.

Baseline Characteristics	Sinus Rhythm (n = 74)	Atrial Fibrillation (n = 31)	p-value
Age, (yrs)	62.5 ± 11.5	66.0 ± 10.5	0.129
Male Sex, n (%)	56 (82.3)	25 (80.6)	0.683
Persistent AF, n (%)	31 (45.6)	26 (83.9)	<0.001
Previous AF Ablation, n (%)	28 (37.3)	8 (25.8)	0.361
AF duration (months)	63 (20–140)	52 (31–132)	0.799
**Cardiovascular Risk Factors**			
BMI (kg/m^2^)	28.7 ± 5.3	29.2 ± 4.2	0.627
Weight (kg)	92.8 ± 17.9	90.2 ± 20.6	0.518
Hypertension, n (%)	49 (65.3)	20 (64.5)	1
Diabetes, n (%)	9 (12)	4 (12.9)	1
Previous Stroke, n (%)	5 (6.7)	4 (12.9)	0.443
Coronary Artery Disease, n (%)	5 (6.7)	3 (9.7)	0.670
Obstructive Sleep Apnea, n (%)	25 (33.3)	7 (22.6)	0.461
Current smoker, n (%)	5 (6.7 %)	2 (6.5 %)	0.999
Previous smoker, n (%)	11 (14.9 %)	2 (6.5 %)	0.336
Alcohol Excess (>30 g/week), n (%)	34 (45.3)	12 (38.7)	0.681
CHA_2_DS_2_-Vasc Score	2.0 (1.0–3.0)	2.0 (1.0–3.0)	0.227
**Medications**			
ACEi/ARB	42 (56.0)	14 (58.1)	1
Beta-blocker	33 (44.0)	11 (35.5)	0.553
MRA	7 (9.3)	2 (6.5)	1
Antiarrhythmic	57 (76.0)	23 (74.2)	1
Loop diuretics	1 (1.5)	5 (16.1)	0.007

### 3.2 Cardiopulmonary exercise testing

All 105 participants included in the final analysis satisfactorily completed the CPET protocol achieving maximal effort defined as an RER > 1.05 and volitional exhaustion. There were no major cardiac events or sustained ventricular arrhythmias during CPET. Overall, mean VO_2Peak_ in the entire cohort was 21.3 ± 7.4 ml/kg/min with 45 (42.9 %) meeting objective criteria for reduced exercise tolerance (VO_2Peak_ < 20 ml/kg/min).

**Fig. 2:**
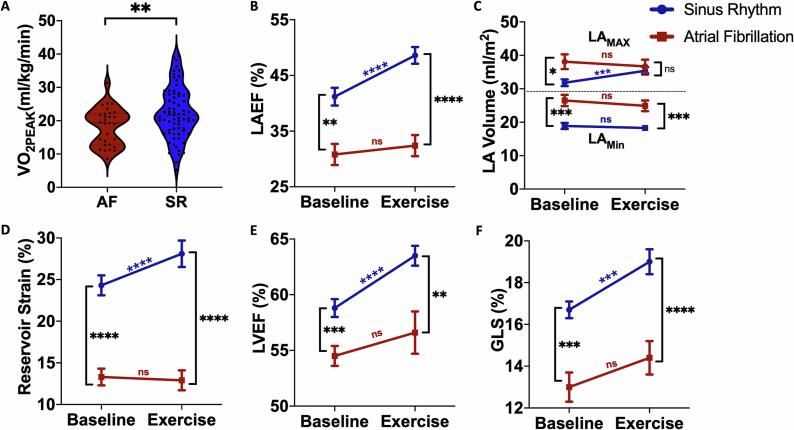
Atrial fibrillation versus sinus rhythm – cardiorespiratory fitness and LA and LV parameters. (A) AF rhythm was associated with reduced VO_2Peak_ compared to patients in SR. (B) LAEF was higher in patients in SR at rest and increased significantly with exercise whereas AF patients did not augment LAEF with exercise. Underpinning these differences in LAEF response was (C) reduced LA_max_ dilatation patients in in AF compared to SR. Exercise did not affect LA_MIN_ in either group. (D) Patients in SR also demonstrated LA reservoir strain reserve with exercise, which was absent in AF. (E) Similarly, LVEF also significantly increased with exercise in patients in SR but not in patients in AF whilst (F) LV GLS was reduced in patients in AF at baseline and did not augment with exercise compared to patients in SR.

Comparing AF with SR, participants presenting in AF had significantly lower cardiopulmonary reserve, demonstrating reduced VO_2Peak_ (18.4 ± 5.6 vs 22.5 ± 7.7 ml/Kg/min, p = 0.003, Fig. 2**A**) and percent of predicted VO_2_ (82.7 ± 18.7 vs 96.4 ± 25.5 %, p = 0.003). Of patients presenting in AF, 17 (54.8 %) demonstrated reduced exercise tolerance whilst 28 (37.8 %) of patients in SR had reduced exercise tolerance (p = 0.165). Patients in AF had higher resting heart rates (83.8 ± 15.2 vs 64.4 ± 12.1 bpm, p < 0.001) but there was no difference in maximal heart rate achieved during CPET (139.5 ± 35.0 vs 133.2 ± 26.2 bpm, p = 0.390) or overall chronotropic response (59.4 ± 33.3 vs 66.4 ± 26.6 bpm, p = 0.34).

### 3.3 Resting and exercise LA function

Table 2 shows the resting and exercise echocardiography results according to presenting rhythm. At rest, patients in AF demonstrated reduced LAEF and reservoir strain. With exercise, LAEF failed to augment amongst those in AF but significantly increased in those presenting in SR (Fig. 2**B**). Overall LAEF reserve (change in LAEF with exercise) was significantly higher with SR compared with AF (+6.9 ± 9.0 vs + 0.7 ± 10.0, p = 0.009). Volumetric LA analysis revealed that participants in AF had larger LA volumes at rest but there were no significant differences in LA_MAX_ or LA_MIN_ with exercise. Participants in SR, on the other hand, demonstrated significant LA_MAX_ dilatation but no difference in LA_MIN_ with exercise (Fig. 2**C**). Similarly, LA reservoir strain was significantly reduced at rest in patients with AF and failed to augment with exercise (Fig. 2**D**) with LA reservoir strain reserve (change in reservoir strain with exercise) significantly higher in the SR group (+4.1 ± 7.3 vs −0.6 ± 4.6 %, p = 0.002).

**Table 2:** Resting and Exercise Echocardiographic Parameters. Left atrial and left ventricular size and function at baseline and peak exercise in patients in AF and SR at the time of exercise. Data are means ± standard deviations. **LA** left atrium, **LAEF** left atrial emptying fraction, **LV** left ventricle, **LVEF** left ventricular emptying fraction, **GLS** global longitudinal strain.

	Resting Echocardiography			Exercise Echocardiography		
	SR (n = 74)	AF (n = 31)	p-value	SR (n = 74)	AF (n = 31)	p-value
LAEF	41.2 ± 13.3	30.8 ± 9.3	<0.001	48.6 ± 12.1^a^	32.4 ± 9.6	<0.001
Reservoir Strain	24.3 ± 9.1	13.3 ± 6.8	<0.001	28.1 ± 11.3^a^	12.9 ± 5.7	<0.001
LA_MAX_	31.9 ± 8.5	38.1 ± 11.6	0.027	35.4 ± 9.2^a^	36.7 ± 9.8	0.561
LA_MIN_	19.0 ± 7.3	26.5 ± 8.9	<0.001	18.3 ± 7.0	24.9 ± 8.2	<0.001
LVEF	58.8 ± 5.9	54.5 ± 4.0	<0.001	63.5 ± 7.0^a^	56.6 ± 7.8	0.001
LV GLS	16.7 ± 3.3	13.0 ± 2.8	<0.001	19.0 ± 3.8^a^	14.4 ± 2.4	<0.001
LV_MAX_	98.5 ± 30.9	84.0 ± 29.7	0.049	98.1 ± 32.3	87.6 ± 25.3	0.183
LV_MIN_	41.3 ± 14.8	41.4 ± 13.0	0.965	36.3 ± 14.5^a^	40.6 ± 14.4	0.301
E/E′	9.2 ± 3.2	9.9 ± 3.6	0.332	9.4 ± 3.5	11.3 ± 6.7	0.247

a p < 0.05 vs SR group at rest.

### 3.4 Resting and exercise LV function

Compared to patients in SR, patients in AF demonstrated significantly reduced LVEF and GLS at rest. During exercise, neither LVEF nor GLS augmented amongst patients in AF but there was significant augmentation amongst patients in SR (Fig. 2**E and 2F**). Underpinning these differences in LV function with exercise was a reduction in LVESV during exercise amongst patients in SR that was not observed amongst those in AF. In terms of diastolic LV function, E/e′ was no different between AF and SR and we did not observe any statistically significant differences in E/e′ between rest and exercise in either group.

### 3.5 Association between LA function and exercise capacity

**Fig. 3:**
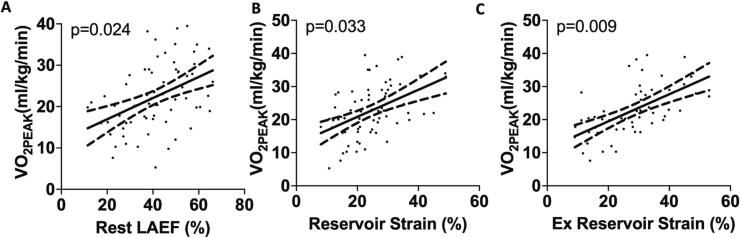
Relationship between LA function and cardiorespiratory fitness in patients in sinus rhythm. (A) Higher LAEF is associated with significantly associated with increased VO_2Peak_ independent of age, gender and resting left ventricular ejection fraction. Similarly, higher LA reservoir strain was associated with higher VO2Peak both (B) at rest and (C) during exercise independent of age, gender and resting left ventricular ejection fraction. **LAEF** left atrial emptying fraction.

Of the 74 patients presenting in SR, 28 (38.7 %) demonstrated objective evidence of reduced exercise capacity (VO_2Peak_ < 20 ml/kg/min). Table 3 shows the association between resting and exercise LA parameters and VO_2Peak_ in the 74 patients presenting in SR. Whilst there was no relationship between VO_2Peak_ and LA volumes, VO_2Peak_ demonstrated significant associations with resting LAEF, reservoir strain and booster strain in model 1. VO_2Peak_ was also significantly associated with LAEF and reservoir strain during exercise in model 1. In model 2, VO_2Peak_ remained significantly associated with resting LAEF (Fig. 3**A**) and reservoir strain (Fig. 3**B**) in addition to reservoir strain during exercise (Fig. 3**C**).

**Table 3:** Relationship between LA parameters and VO_2Peak_ in patients in SR. Adjusted linear regression models to assess the relationship between LA size and function and exercise capacity. **LA** left atrium, **LAEF** left atrial emptying fraction. Bold indicates statistical significance.

Variable	Model 1*		Model 2**	
	Coefficient (95 % CI)	p-value	Slope (95 % CI)	p-value
**Resting LA Parameters**				
LA_max_ (ml/m^2^)	0.04 (−0.15 to 0.23)	0.655	0.09 (−0.13 to 0.31)	0.414
LA_min_ (ml/m^2^)	−0.17 (−0.41 to 0.07)	0.169	−0.16 (−0.43 to 0.12)	0.26
**LAEF (%)**	**0.14 (0.02 to 0.27)**	**0.028**	**0.17 (0.02 to 0.31)**	**0.024**
**Reservoir Strain (%)**	**0.26 (0.07 to 0.45)**	**0.007**	**0.25 (0.02 to 0.49)**	**0.033**
**Booster Strain (%)**	**0.34 (0.05 to 0.62)**	**0.021**	0.31 (−0.03 to 0.65)	0.076
Conduit Strain (%)	0.30 (−0.01 to 0.62)	0.056	0.33 (−0.06 to 0.73)	0.098
**Exercise LA Parameters**				
LA_max_ (ml/m^2^)	0.11 (−0.06 to 0.28)	0.211	0.11 (−0.08 to 0.30)	0.245
LA_min_ (ml/m^2^)	−0.05 (−0.30 to 0.21)	0.713	−0.04 (−0.31 to 0.23)	0.761
**LAEF (%)**	**0.16 (0.0006 to 0.32)**	**0.049**	0.15 (−0.02 to 0.32)	0.084
**Reservoir Strain (%)**	**0.31 (0.11 to 0.51)**	**0.003**	**0.31 (0.08 to 0.54)**	**0.009**

* Model 1 – adjusted for age and sex.

** Model 2 – adjusted for age, sex and resting LVEF.

## 4 Discussion

### 4.1 Major findings

This prospective clinical study utilised exercise echocardiography and CPET to investigate LA function during exercise and its association with exercise intolerance in patients with symptomatic AF. The study has identified several novel findings:

1. Amongst AF patients in SR, the LA response to exercise is characterised by an augmentation of LA emptying fraction, primarily through an increase in LA filling, and a concomitant increase in LA reservoir strain.
2. In contrast, patients in AF demonstrate reduced LA and LV function at rest and a failure to augment LA emptying and LV function with exercise. These differences are observed in parallel with reduced exercise capacity on CPET.
3. A high proportion (37.8 %) of AF patients presenting in SR exhibit objective evidence of reduced exercise capacity. Reduced exercise capacity in AF patients maintaining SR is associated with reduced resting and exercise LAEF and reservoir strain, independent of LV function.

### 4.2 Atrial response to exercise in sinus rhythm

The influence of AF on LA mechanical function at rest has been well described using transthoracic echocardiography and cardiac magnetic resonance imaging. Our study extends on previous findings by using exercise echocardiography to evaluate the LA response to exercise, which has not been well described during exercise, AF patients in SR demonstrate a capacity to increase LAEF, primarily due to an increase in LA_max_, with stable LA_min_. The increase in LAEF is frequently observed during exercise amongst healthy individuals. However, there is limited data on the atrial response to exercise in AF patients. This study demonstrates that, despite the presence of underlying atrial disease promoting arrhythmia, AF patients in SR retain the capacity to increase the atrial contribution to LV filling during exercise. Notably, our data show that LAEF is enhanced through an increase in LA_max_ in the absence of a reduction in LA_min_. This pattern of response is consistent with that shown in healthy participants without AF [10]. In addition to increased LAEF, we also demonstrated an LA strain reserve during exercise in patients who were in SR, consistent with that observed elsewhere in the presence of HF [11].

### 4.3 Atrial response to exercise in AF

With the loss of atrial systole during AF, the atrial contribution to the cardiac response during exertion is frequently overlooked. We provide novel information on the atrial contribution to LV filling amongst patients in AF at the time of assessment. As expected, patients in AF had larger LA size at rest and reduced LA function, both on volumetric and strain measures. In contrast to patients in SR, those in AF showed little ability to augment LAEF or LA strain with exercise. The limited ability to dilate the LA with the onset of exercise may contribute to the lower exercise tolerance observed with AF, in addition to the loss of LA contraction. We also demonstrated a limited LV response to exercise with a blunted LVEF response and GLS reserve. We speculate that the absence of LA contraction may limit LV filling and preload, subsequently limiting stroke volume [12], [13], [14], [15], [16]. However, it may also be that reduced LV contraction limits the apical movement of the atrio-ventricular plane, limiting the aspiration of blood from the pulmonary veins into the LA. Overall, we show that patients presenting in AF rhythm demonstrate significantly reduced exercise capacity on CPET compared to those presenting in SR, despite no difference in exercise heart rate or underlying risk factors. Based on this data, we attribute this limited exercise capacity, in part, due to restricted augmentation of LA filling, in addition to the absence of atrial contraction. Our data therefore highlights the influence AF rhythm independent of rate control on cardiac function during exercise and overall exercise capacity. We also provide further mechanistic evidence to support previous findings that exercise capacity may be improved through rhythm control strategies including DC cardioversion [17], [18], [19], antiarrhythmic drugs [20] and AF ablation [21], [22].

### 4.4 LA function and exercise capacity in sinus rhythm

Of those presenting in SR, we found that more than one third demonstrated objective evidence of reduced exercise capacity according to the Weber Classification System [9]. This finding highlights the fact that for many AF patients, exercise intolerance is related to factors beyond rhythm control, as has previously been described [4]. Our linear regression analysis suggests that LA function at rest and during exercise are strongly associated with exercise capacity in these patients.

The healthy LA contributes 15–30 % of overall LV stroke volume [23] and it is known that the loss of atrial activity characterised by the rhythm of AF results in reduced cardiac output during exercise [24]. However, exercise intolerance is frequently reported in patients who maintain SR and may be the consequence of underlying atrial disease mechanisms. In this study, we demonstrate that reduced LA reservoir and emptying function in SR is associated with reduced exercise capacity, independent of LV function. This findings mirrors that from patients HF across a broad range of ejection fraction [7], [11]. It is well-established that AF patients demonstrate impaired LA function and LA strain in SR and these changes have been shown to be associated with important prognostic effects including mortality and stroke [23], [24]. However, the relationship between LA function and exercise capacity in patients with AF has not been established. Furthermore, to our knowledge, this is the first study to correlate LA function during exercise with objective assessment of exercise capacity. Importantly, we show that impaired LA function during exercise also correlates closely with reduced exercise capacity, confirming the likely contribution of LA function to overall cardiac output during exercise.

### 4.5 Atrial dysfunction and the interaction between AF and HF

Whilst LA mechanical dysfunction is an established hallmark of AF, it is increasingly recognised as an important feature of heart failure with preserved ejection fraction (HFpEF). Growing evidence suggests that AF and HFpEF are closely related and commonly coexist due to the presence of atrial dysfunction [25], [26]. In heart failure (including reduced and preserved ejection fraction), it has been shown that impaired LA function is associated with reduced stroke volume and cardiac output at peak exercise [27]. Our findings that LA dysfunction is closely related to exercise intolerance in patients with AF and preserved LVEF strengthens the likelihood that ‘early’ HFpEF may be an underlying feature of exercise intolerance in symptomatic AF. It therefore follows that treatment of underlying HFpEF may result in improvements in exercise capacity for these patients. Interestingly, a recent small randomised trial suggested that AF ablation for patients with AF and haemodynamically confirmed HFpEF resulted in significant improvements in invasive haemodynamics compared to medical therapy and this was associated with improvements in maximal exercise capacity [28]. In addition, there is evidence that exercise training can improve LA function resulting in exercise capacity gains in heart failure with mid-range ejection fraction patients [29]. There is further promise in the development of SGLT2-inhibitors as a treatment for HFpEF [30], [31]; future work should investigate the effect of lifestyle changes and medications on cardiac function and exercise tolerance in patients with AF.

### 4.6 Limitations

We recognise several limitations that should be considered when interpreting this study. We used 2D transthoracic echocardiography, which may not provide the accuracy of exercise cardiac magnetic resonance imaging (CMR). Validation of these findings with exercise CMR should be considered. The absence of direct assessment of stroke volume or invasive haemodynamic limits our interpretation of the response to exercise. Studies in which simultaneous assessment of invasive pressures and imaging would be an advantage in this setting. We did not compare patients in AF after reversion to sinus rhythm. Therefore, these findings do not provide evidence that restoration of sinus rhythm would restore LA function during exercise. Likewise, we did not assess other features of atrial disease, such as fibrosis or electroanatomical remodelling between groups, which may influence the atrial response to exercise. Our sample size was relatively small, which may open the possibility for type II error in comparing between and within-groups. In addition we did not compare the atrial response to exercise with non-AF control subjects, However, the atrial response to exercise in peoplewithout AF has been described previously [10], [27].

## 5 Conclusions

In patients with symptomatic AF, the maintenance of sinus rhythm is associated with preserved capacity to augment LA filling and function during exercise. In contrast, LA reservoir function is limited at rest and during exercise amongst patients in AF at the time of assessment. These divergent responses parallel a reduced VO_2peak_ amongst patients in AF. Amongst patients who are in SR, exercise intolerance was observed in 39 % of patients. Exercise intolerance amongst AF patients maintaining SR is associated with reduced LA reservoir strain and emptying fraction at rest and during exercise, independent of resting or exercise LV function. Taken together our data highlight the potential role of LA function in contributing to maximal exercise capacity in patients with AF. Future research investigating the influence of novel therapies for AF on LA function at rest and during exertion may improve our understanding of the effect of these therapies on patient symptoms and functional capacity.

## CRediT authorship contribution statement

**Jonathan P. Ariyaratnam:** Data curation, Formal analysis, Investigation, Writing – original draft. **Ricardo S. Mishima:** Investigation, Methodology, Writing – review & editing. **Olivia McNamee:** Investigation, Methodology, Writing – review & editing. **Mehrdad Emami:** Investigation, Methodology, Writing – review & editing. **Anand Thiyagarajah:** Investigation, Writing – review & editing. **John L. Fitzgerald:** Investigation, Writing – review & editing. **Celine Gallagher:** Methodology, Supervision, Writing – review & editing. **Prashanthan Sanders:** Conceptualization, Supervision, Writing – review & editing. **Adrian D. Elliott:** Conceptualization, Formal analysis, Funding acquisition, Investigation, Methodology, Supervision, Writing – review & editing.

## Declaration of competing interest

The authors declare that they have no known competing financial interests or personal relationships that could have appeared to influence the work reported in this paper.
